# Impact of Body Mass Index on Tumor Recurrence in Patients Undergoing Liver Resection for Perihilar Cholangiocarcinoma (pCCA)

**DOI:** 10.3390/cancers13194772

**Published:** 2021-09-24

**Authors:** Hans-Michael Hau, Mareen Devantier, Nora Jahn, Elisabeth Sucher, Sebastian Rademacher, Daniel Seehofer, Robert Sucher

**Affiliations:** 1 Department of Visceral, Transplantation, Vascular and Thoracic Surgery, University Hospital of Leipzig, 04103 Leipzig, Germany; hans-michael.hau@uniklinikum-dresden.de (H.-M.H.); mareen.devantier@gmail.com (M.D.); sebastian.rademacher@medizin.uni-leipzig.de (S.R.); daniel.seehofer@medizin.uni-leipzig.de (D.S.); 2 Department of Visceral, Thoracic and Vascular Surgery, University Hospital Carl Gustav Carus, Technische Universität Dresden, 01307 Dresden, Germany; 3 Department of Anesthesiology and Intensive Care Medicine, University Hospital of Leipzig, 04103 Leipzig, Germany; nora.jahn@medizin.uni-leipzig.de; 4 Department of Oncology, Gastroenterology, Hepatology, Pneumology, Infectiology, and Nutritional Medicine, University Hospital of Leipzig, 04103 Leipzig, Germany; elisabeth.sucher@medizin.uni-leipzig.de

**Keywords:** perihilar cholangiocarcinoma, liver resection, recurrence, body mass index, obesity, prognosis

## Abstract

**Simple Summary:**

Perihilar cholangiocarcinoma (pCCA) is a relatively rare and aggressive hepatobiliary tumor with a general poor prognosis. Surgical therapy remains the only curative treatment option with the best prospects for long-term survival. However, tumor recurrence is frequent, and is associated with a poor prognosis. The identification of risk factors as well as appropriate selection of surgical candidates is essential to accurately predict prognosis and to maximize survival while decreasing tumor recurrence rates. Previous studies have already established a link between an increased BMI and the occurrence of various tumors. At present, data on BMI-associated long-term outcome following curative liver resection in pCCA patients are warranted. This study aims to investigate the impact of increased BMI on patient’s outcome, especially on tumor recurrence, following liver resection in patients with pCCA as well as to evaluate prognostic and risk factors for accurate prediction of outcome in this selective group of patients.

**Abstract:**

Background: The association of body mass index (BMI) and long-term prognosis and outcome of patients with perihilar cholangiocarcinoma (pCCA) has not been well defined. The aim of this study was to evaluate clinicopathologic and oncologic outcomes with pCCA undergoing resection, according to their BMI. Methods: Patients undergoing liver resection in curative intention for pCCA at a tertiary German hepatobiliary (HPB) center were identified from a prospective database. Patients were classified as normal weight (BMI 18.5–24.9 kg/m^2^), overweight (BMI 25.0–29.9 kg/m^2^) and obese (>30 kg/m^2^) according to their BMI. Impact of clinical and histo-pathological characteristics on recurrence-free survival (RFS) were assessed using Cox proportional hazard regression analysis among patients of all BMI groups. Results: Among a total of 95 patients undergoing liver resection in curative intention for pCCA in the analytic cohort, 48 patients (50.5%) had normal weight, 33 (34.7%) were overweight and 14 patients (14.7%) were obese. After a median follow-up of 4.3 ± 2.9 years, recurrence was observed in totally 53 patients (56%). The cumulative recurrence probability was higher in obese and overweight patients than normal weight patients (5-year recurrence rate: obese: 82% versus overweight: 81% versus normal weight: 58% at 5 years; *p* = 0.02). Totally, 1-, 3-, 5- and 10-year recurrence-free survival rates were 68.5%, 44.6%, 28.9% and 13%, respectively. On multivariable analysis, increased BMI (HR 1.08, 95% CI: 1.01–1.16; *p* = 0.021), poor/moderate tumor differentiation (HR 2.49, 95% CI: 1.2–5.2; *p* = 0.014), positive lymph node status (HR 2.01, 95% CI: 1.11–3.65; *p* = 0.021), positive resection margins (HR 1.89, 95% CI:1.02–3.4; *p* = 0.019) and positive perineural invasion (HR 2.92, 95% CI: 1.02–8.3; *p* = 0.045) were independent prognostic risk factors for inferior RFS. Conclusion: Our study shows that a high BMI is significantly associated with an increased risk of recurrence after liver resection in curative intention for pCCA. This factor should be considered in future studies to better predict patient’s individual prognosis and outcome based on their BMI.

## 1. Introduction

Since 1975, the prevalence of obesity has nearly tripled worldwide [1,2]. With regard to a report by the world health organization (WHO) in 2016, more than 1.9 billion (39%) of adults with age of 18 years and older, were overweight and more than 650 million (13% of the adults world population) were obese [2]. Concerning the European Region (EU), these numbers are even higher in relation to patients being overweight with a prevalence of 59% and a prevalence of obesity of 23% of all adults.

The increasing number of obesity represents a major public health problem and in this context obesity-associated cancer imposes a large economic burden [3,4]. According to various pervious reports, obesity and overweight could be identified as independent risk factors for many type of gastrointestinal and genito-uterinary cancers including cholangiocarcinoma (CCA) [5,6].

Although the perihilar cholangiocarcinoma (pCCA) represents the fourth most common gastrointestinal tumor, pCCA is a relatively rare and aggressive hepatobiliary tumor with a general poor prognosis [7,8]. Surgical therapy remains the only curative treatment option with the best prospects for long-term survival in this patient group. Nevertheless, surgical treatment in the case of patients with pCCA is a difficult, complex and technically challenging undertaking [9,10]. In the last three decades, the extent of surgical resection has changed to a more aggressive procedure. Specifically, this included resection of the extrahepatic bile duct combined with a major hepatectomy, en-bloc resection of the caudate lobe and extensive lymph node dissection such as vascular resection and reconstruction of the portal vein and/or hepatic artery when indicated [7,11]. Unfortunately, most of the patients with pCCA show metastatic or locally advanced disease at first clinical presentation, and despite an aggressive surgical approach with curative intention, many patients develop tumor recurrence. Survival after curative liver resection is mediocre with 5-year overall survival rates varying from 25% to 40% according to recent studies [7,9,12,13,14]. Poor long-term survival rates of patients with pCCA predominantly result from high and early recurrence rates. Better understanding and knowledge of recurrence and recurrence patterns may improve therapeutic strategies as well as intraoperative and postoperative decision making.

Several studies analyzed and identified prognostic risk factors for the development of pCCA in term of its prognosis and recurrence [13,15,16,17,18,19]. However, data reflecting the association of recurrence and obesity in pCCA are limited. The current literature contains few studies that have analyzed the impact of elevated/reduced body mass index (BMI) and long-term oncological outcome for patients undergoing liver resection for intrahepatic cholangiocarcinoma (iCCA) [20,21]. However, to the best of our knowledge no study investigated the effects of obesity on prognosis and oncological outcome for patients undergoing curative liver resection for pCCA.

In this light, the objective of the current study was to investigate the impact of increased BMI on patient’s outcome, especially on tumor recurrence, after liver resection in patients with pCCA. Further, we also aimed to evaluate prognostic and risk factors with regard to recurrence-free survival in this selective group of patients.

## 2. Material and Methods 

### 2.1. Study Population

This study included data of a prospective database from patients undergoing liver resection for pCCA with curative intention. The study was approved by the local Ethics Committee of the University Hospital of Leipzig (AZ EK: 243-14-14072014).

Only patients who had undergone curative liver resection and had histologic confirmation of their pCCAs as defined by the guidelines of American Joint Committee on Cancer (AJCC) or International Union Against Cancer (UICC) by were included in the study. For tumor grading, the Bismuth Corlette classification was used [22].

According to the above definitions for PCA, only patients with tumors “involving the hilar bile duct (defined as the duct located topologically between the right side of the umbilical portion of the left portal vein and the left side of the origin of the right posterior portal vein)” were included into our analysis [23,24,25].

Patients who had suffered in-hospital mortality/postoperative mortality within 30 days, who underwent a palliative (R2) resection and those who had undergone ablation therapy were excluded from further analyses. In addition, patients with missing information about their BMI status and any specific factor included in the multivariable model as well as a final pathology indicative of a diagnosis other than pCCA were also excluded.

At hospital admission, participants body mass index were recorded before surgery according to the usual formula: weight (kg)/height (m) squared [26]. According to the WHO classification, patients were initially categorized as normal weight (BMI: 18.5–24.9 kg/m^2^), overweight (25–29.9 kg/m^2^) and obese >30 kg/m^2^.

### 2.2. Preoperative Patient Management 

A standardized preoperative diagnostic workup was performed, and a multidisciplinary decision was obtained within a tumor board conference. This staging included besides systemic inspection and physical examination of the patient as well as extensive cross-sectional imaging examinations including computed tomography (CT) of the abdomen and chest, as well as magnetic resonance imaging (MRI) with magnetic resonance cholangiopancreatography (MRCP) of the liver. Patients who had clinically and/or radiologically an obstructive jaundice and/or cholangitis beforehand were treated with a biliary drainage, preferably into the future liver remnant (FLR). The endoscopic procedure as Endoscopic biliary drainage (EBD) is the method of choice, whereas percutaneous transhepatic biliary drainage (PTBD) was only performed in unsuccessful cases. By default, brush cytology was taken as part of the preoperative endoscopy for drain placement, but confirmation of malignancy in this was not considered as a “conditio sine qua non” to proceed with surgery. If preoperative imaging was unclear and/or peritoneal carcinomatosis was suspected, diagnostic laparoscopy was performed. In patients who are suspected of having an insufficient size and function of the FLR, preoperative portal vein embolization (PVE) was performed. The augmentation of the FLR was checked prior liver resection by CT volumetry and volumes >25% of the total liver volume were considered sufficient.

However, unless specified elsewhere, surgery was not performed in cases of poor patient’s condition, restricted liver function, present extensive bilobular metastases or extrahepatic metastases, peritoneal carcinomatosis as well as infiltration and/or encasement of major vascular structures without the possibility for resection and reconstruction.

### 2.3. Surgical Procedure

Our nomenclature of hepatic anatomy and resection was in compliance with the Brisbane 2000 classification system [27]. Accordingly, we performed a series of anatomic left and right hemihepatectomies with or without wedge resections of adjacent liver segments as well as extended left and right hemihepatectomies commonly entitled as trisectionectomy. The operative procedure included a case dependent en-bloc resection of segment 1 as well as a dissection of lymph node groups 1, 2 and 3 as described earlier [9,28,29].

The exact extent and method of liver resection depends on the location of the tumor, its extent and depth of infiltration, which is classified according to the Bismuth–Corlette classification [30,31].

This includes that in the case of vascular infiltration, resection with consecutive reconstruction of the portal vein and hepatic artery was performed.

### 2.4. Follow-Up/Surveillance

The follow-up care after liver resection for PHC in our center was carried out according to a strict and standardized follow-up protocol. This involves patients’ presentation of the following standardized steps including physical examination, abdomen ultrasonography and laboratory control every 3 months in the first year after resection, then every 6 months in the second and third year and then annually. During the laboratory check, the tumor markers such as carcinoembryonic antigen (CEA) and cancer antigen 19-9 (CA 19-9) as well as standard liver laboratory values are always routinely taken. Furthermore, after 6, 12, 24 and 36 months, as well as in the case of unclear sonographic findings/unclear clinical findings with a suspicion of recurrence/ increased tumor markers a sectional imaging examination consisting of MRI with MRCP and CT thorax is routinely performed.

### 2.5. Data Collection and Variables

From our prospectively maintained clinical database following data were collected and analyzed for each patient: 

Demographic characteristics and clinic-pathological parameters including age, gender, American Society of Anesthesiologists (ASA) classification, jaundice (with preoperative bilirubin levels), tumor marker status (Serum levels of carcinoembryonic antigen (CEA), Cancer Antigen (CA) 19-9) and medical history (presence of liver cirrhosis, diabetes mellitus). We also collected preoperative treatment characteristics and operative details including length of hospital stay/intensive care unit stay, administration of neoadjuvant and adjuvant chemotherapy, type and extent of liver surgery, use of blood transfusion, preoperative drainage/endoscopic stenting and previous PVE such as bile duct involvement (according the Blumgart and Bismuth–Corlette classification) and vascular involvement by imaging.

For the analysis of pathological and tumor-specific characteristic data, the tumor size, histological grading, resection margin status (R0-microscopically negative (R0) or microscopically positive (R1)), location and total amount of metastasic lymph nodes as well as the number of harvested lymph nodes were recorded. Further pathological assessment included knowledge about perineural, lymphvascular and biliary invasion of the pCCA.

Knowledge of the direct infiltration/invasion of portal vein, caudate lobe, hepatic artery, and contiguous organs are another important collected tumor characteristic.

Pathologic tumor staging of the PHC based on the newest 8th TNM classification of the UICC and AJCCC [23,24].

Collected short- and long-term outcomes after liver resection included peri- and postoperative complications (assessed according to the Clavien–Dindo Classification [32]), recurrence free survival such as recurrence rates.

As reported previously, recurrence was defined by suspicious findings based on imaging (CT, MRI) or proven by a tumor biopsy [17].

According to previous definitions, recurrence was classified as either locoregional or distant. With regard to the newly 8th AJCC classification, the term “locoregional” was newly introduced defining as any recurrence at the hepaticojejunostomy, at the liver resection margin, distal bile duct remnant or elsewhere surgical procedures have been performed including liver hilum, hepatoduodenal ligament, common hepatic artery, and peripancreatic lesions [16,17].

All other recurrences were defined as distant.

### 2.6. Statistical Analysis

Continuous variables were expressed as mean values with standard deviation (SD), whereas categorical variables were summarized as total counts and percentages (%).

In accordance with general practice, the appropriate test was used for the analysis of the baseline data including chi-square test, Student´s *t*-test, analysis of variance (ANOVA), Kruskal–Wallis and/or Wilcoxon–Mann–Whitney test were applied.

With regard to general previous definitions, the recurrence interval (RI) was as usually measured form the date of liver surgery to the first occurrence of recurrence.

In this context, patients who had not developed recurrent disease were censored at the time of last follow-up. While patients who died due to other causes were censored at the time point of death. Recurrence-free survival (RFS) was defined by the time from liver surgery to the first detection of recurrence or patient’s death from any cause.

Patients who were alive without any recurrence were censored at the time of last follow-up. For survival analysis the Kaplan–Meier method was used, and a log-rank test was applied to check significance of the different survival in univariable analysis. Variables with *p* < 0.05 in the univariable analysis were entered in a stepwise Cox proportional hazard regression model for multivariate analysis to check their independency. Results were presented as hazard ratios (HRs) with 95% confidence intervals (CIs) and *p*-values. SPSS software (SPSS Inc., Chicago, IL, USA, version 25) was used for data analysis and a *p*-value < 0.05 was considered as statistically significant.

## 3. Results

### 3.1. Patient Characteristics and Preoperative Data 

Between 1999 and 2019 a total of 211 consecutive patients were evaluated and underwent surgery with respect to PHC at our center. Of these, 116 patients were excluded, including 97 patients with pM1 disease, four with extensive liver disease (liver fibrosis/reduced remaining liver function/capacity), five with R2 resection, seven who died in hospital with postoperative complications (7.3%) as well as three with no detailed follow up information. The remaining 95 patients (45%) were enrolled in the remaining study and liver resection was performed with curative intention.

In totally, 95 patients (45%) met the inclusion criteria and proceeded to liver resection with curative intention.

Table 1 summarizes the baseline characteristics and preoperative data of our patient collective.

Of these, 48 patients (50.5%) had normal weight, 33 (34.7%) were overweight and 14 patients (14.7%) were obese. Mean BMI in the normal, overweight and obese group was 22.6 ± 1.69 kg/m^2^, 27.5 ± 1.4 kg/m^2^ and 33.8 ± 3.4 kg/m^2^, respectively (*p* < 0.01).

Preoperative weight loss of more than 10% of initial body weight within 3-month prior surgery was recorded in 9 patients (normal weight: 5 (10%), overweight: 3 (9%) and obese: 1 (7%); *p* = 0.93) which was equally distributed among cohorts.

Mean patient age, sex and preoperative weight loss showed no significant differences between the three BMI-category groups, whereas normal weight patients had lower ASA scores than overweight and obese patients (ASA 1–2 normal weight: 82%, overweight: 70% and obese: 36%; *p* < 0.01).

There were no significant differences regarding pre-existing medical conditions like presence of diabetes mellitus (27% versus 24% versus 42%; *p* = 0.21), liver cirrhosis/fibrosis (10% versus 12% versus 14%; *p* = 0.91) as well as preoperative jaundice (79% versus 82% versus 79%; *p* = 0.79) among normal, overweight and obese patients.

Preoperative laboratory chemical examinations showed a mean CEA and CA 19-9 value of 4.3 ± 8.2 and 318 ± 540 U/L, respectively, without significant differences between the BMI- groups.

With regard to the Bismuth–Corlette classification, type IV (*n* = 49; 51.6%) pCCAs are the most common ones. They are followed by six patients (6.3%) with type I, eight patients (8.4%) with type II and 23 patients (24.2%) having type III pCCAs. A preoperative biliary drainage was placed in 72 patients without significant differences within all BMI categories (*p* > 0.05). In total, nine patients (9.5%) received neoadjuvant chemotherapy.

### 3.2. Tumor Characteristics

Tumor characteristics of the study population according to their BMI are displayed in Table 2. Negative resection margins (R0) could be achieved in 68 patients (71.6%), whereas 27 patients (29.4%) had a R1-resection. Overall, only slight differences in tumor characteristics were observed among patients in the normal, overweight and obese BMI- categories. Normal weight patients presented with a larger tumor size when compared to obese and overweight patients (*p* = 0.09). Other pathological characteristics like tumor grading, microvascular invasion, perineural invasion and lymphatic vessel invasion could be observed without significant differences among patients within the three BMI groups (all *p* > 0.05).

Furthermore, AJCC 8th edition T and N stages were comparable between normal, overweight and obese patients (*p* > 0.05). According to the 8th AJCC TNM classification, five patients (5.3%) were stage I, 52 patients (54.7%) were stage II, 27 (28.4%) were stage III and eleven patients (11.6%) were classified as stage IV. Finally, mean number of harvested lymph nodes and mean number of lymph nodes showing metastasis were similar among the different BMI categories (all *p* > 0.05).

### 3.3. Peri-Operative and Postoperative Outcomes

Overall, differences in perioperative and operative outcomes of patients are shown in Table 3. Hepatic resections comprised eleven (12%) left hepatectomies, ten (11%) right hepatectomies, ten (11%), 16 (17%) left and 54 (57%) right trisectionectomies, and four (4%) mesohepatectomies all equally distributed between the three BMI groups. Portal vein resection was performed in 64 patients (67.3%) showing no significant differences between the BMI groups (*p* > 0.05). Mean operative times, mean blood loss and the perioperative need for blood transfusions were similar among obese, overweight and normal weight patients (*p* > 0.05). The mean length of hospital stay was 27.6 ± 6.6 days, the mean stay in ICU was 7.1 ± 6.6 days, respectively.

In total, postoperative complications were observed in 66 cases (69%) with 55% of major complications (>grade III according to Clavien-Dindo). Both the incidence of postoperative complications such as the grade of minor and major complications were comparable between obese, overweight and normal weight patients (*p* > 0.05). However, significant more obese patients (*n* = 5; 35.7%) received adjuvant chemotherapy when compared to overweight (*n* = 9; 27.3%) and normal weight patients (*n* = 4; 8.3%) (*p* = 0.02).

### 3.4. Recurrence

During the median follow-up time of 4.3 ± 2.9 years, 53 patients (56%) were diagnosed with disease recurrence. The overall median time to recurrence was 27 (CI: 20.8–38.1) months. Of these 53 patients, 11 (78%) patients were obese whereas recurrence could be observed in 21 (64%) and 21 (44%) among overweight and normal weight patients (*p* = 0.03).

**Figure 1:**
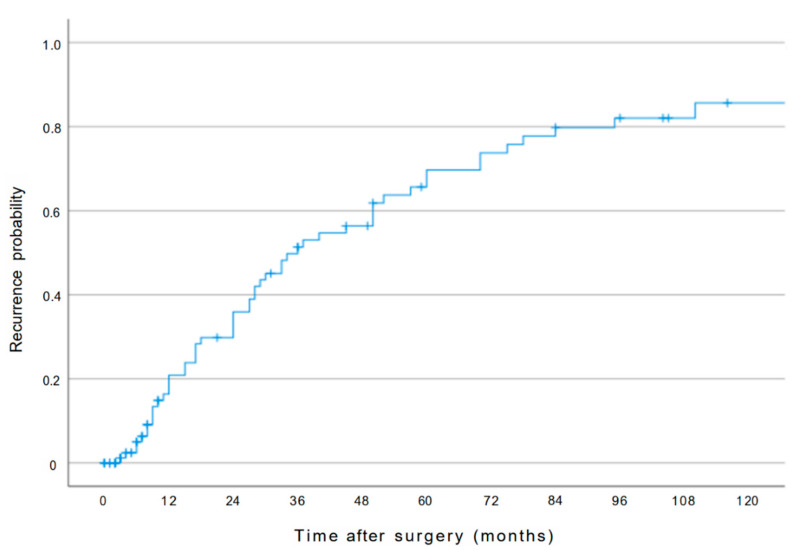
Overall cumulative recurrence curve of patients following liver resection for pCCA.

The 1-, 3-, 5- and 10-year recurrence rates for all patients were 21%, 52%, 69% and 85%, respectively (Figure 1). The estimated cumulative probability of recurrence was significantly higher in the obese group than in the overweight and normal weight group (obese patients: 82% vs overweight patients: 81% vs. normal weight patients: 58% at 5 years, *p* = 0.02).

With regard to location and patterns of recurrence after curative liver resection for pCCA no significant differences could be observed among the three BMI groups (*p* > 0.05). In detail, distant metastatic disease was found in 38.9% (*n* = 37) of patients (obese: 9.5%, *n* = 9 patients vs. overweight: 15.8%, *n* = 15 patients vs. normal weight: 13.6%, *n* = 13 patients, *p* = 0.187), while 16.8% (*n* = 16) of patients (obese: 2.2%, *n* = 2 vs. overweight: 7.3%, *n* = 7 vs. normal weight: 7.3%, *n* = 7, *p* = 0.708) experienced locoregional recurrence.

The primary site of distant recurrence was the liver in 10 cases (27%), the peritoneum in 10 cases (27%), retroperitoneal lymph nodes in seven (19%) cases, the lung/mediastinum in five cases (13%), abdominal wall in 2 cases (6%) and others in three cases (8%).

### 3.5. Recurrence-Free Survival Analysis 

During the follow-up period, 63 patients (66%) died. PHC was the cause of death for 58 patients (92%): 53 patients (84%) had a documented recurrence, five patients died from an undocumented and unconfirmed, but also not excludable diagnosis of recurrence or the consequences of PHC/late complications resulting from biliary drainage or surgery. Five patients (8%) died of other causes.

**Figure 2:**
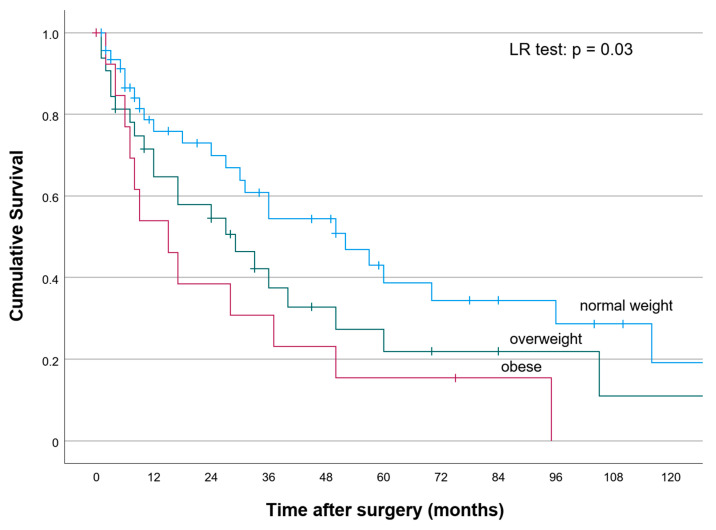
Recurrence-free survival of obese, overweight and normal weight patients following liver resection for pCCA.

The median recurrence-free survival (RFS) for all patients was 31 months (CI:23.4–38.5). The 1-, 3-, 5- and 10-year RFS for the entire study population was 68.5%, 44.6%, 28.9% and 13%, respectively. According to RFS, significant differences were observed among obese, overweight and normal weight BMI categories (Figure 2) (*p* < 0.05). The 1-, 3-, 5- and 10-year RFS was 54%, 36%, 18% and 0% for obese patients versus 67%, 42%, 24% and 12% among overweight patients, and 75%, 54%, 38% and 19%, among normal weight patients (*p* = 0.03), respectively.

Using univariate analysis, several prognostic factors could be identified for poor RFS following curative-intent resection for PHC: age >65 years (*p* = 0.03), positive resection margin (*p* < 0.01), moderate/poor tumor differentiation (*p* < 0.01), positive lymph nodes (*p* = 0.015), vascular invasion (*p* = 0.037), perineural invasion (*p* = 0.021), invasion of small lymphatic vessels (*p* < 0.01), AJCC stage III or IV (*p* = 0.03), perioperative blood transfusion (*p* < 0.01) (Table 4).

Multivariate analysis showed that an increase in BMI (analyzed as continuous variable) per 1 unit was associated with an increased risk of developing recurrence (HR 1.08, 95% CI: 1.01–1.16; *p* = 0.021).

Additionally, multivariate analysis could reveal moderate/poor tumor differentiation (HR 2.49, 95% CI: 1.2–5.2; *p* = 0.014), positive perineural invasion (HR 2.92, 95% CI: 1.02–8.3; *p* = 0.045), positive lymph nodes (HR 2.01, 95% CI: 1.11–3.65; *p* = 0.021) and a positive resection margin (HR 1.89, 95% CI:1.02–3.4; *p* = 0.019) as independent prognostic risk factors for poor RFS (Table 5). All other variables were not associated with RFS in multivariate analysis.

## 4. Discussion

Within the last decade, several studies investigated possible clinicopathological factors associated with prognosis in patients with pCCA treated by liver resection [15,16,17,33,34,35]. However, little is known about the impact and the role of increased body mass index as a prognostic tool among this selected group of patients. In pCCAs, adequate staging and accurate prediction of outcome after liver resection are challenging, and most prognostic and predictive tools and nomograms display poor or only moderate accuracy [18,19,36]. However, systematic estimations of prognostic parameters for short- and long-term outcomes after pCCA resection are essential, especially for the process of peri- and postoperative management as well as for adequate informed consent and shared decision-making.

As such, the current study is important because it is the first to describe the impact of BMI as a strong prognostic parameter for recurrence in patients with pCCA undergoing curative liver resection.

Prediction and prognostic models such as the understanding of risk factors for good and bad prognosis could be helpful to predict accurate prognosis for individualized patients, may refine individualized tumor therapy and support crossover comparison between patients clinical and oncological data. Within the last years, scientific literature showed that the appearance of obesity was associated with increased risk for higher morbidity and mortality in operated patients as well as stronger development of several gastrointestinal, genitourinary and hematological cancers having worse cancer outcome independently of tumor stage and disease presentation [4,6]. When considering patients with primary liver tumors, the current literature and research to date has focused almost exclusively on patients with hepatocellular carcinoma (HCC) and intrahepatic cholangiocarcinoma (iCCA). In this setting, the group by Shinkawa et al. could show that obesity was an independent risk factor for increased HCC recurrence rate in HCV-related patients after preoperative antiviral therapy followed by surgical treatment [37]. A further study by Mathur et al. reported a double increased incidence of recurrence of tumor recurrence in obese patients undergoing liver transplantation due to HCC compared to normal-weight patients [38].

With regard to cholangiocarcinoma, especially in case of pCCAs, little is known about the influence of increased BMI on disease recurrence and recurrence-free survival. In contrast, the group around Merath et al. demonstrated in patients with iCCA that an increasing patients BMI in multivariate analysis is an independent risk factor for increased tumor recurrence after curative liver resection [20].

After surgical resection of pCCAs, the incidence of recurrence can be high ranging from 49% to 76% [15,16,17,33,34,35]. Although, within the least years, some studies investigated recurrence after liver resection for pCCA and identified several pathologic characters associated with worse prognosis, currently available prognostic schemes and nomograms/staging systems showed only limited ability to predict RFS and overall survival among patients with pCCA undergoing curative liver resection [15,16,17,33,34,35,39,40,41,42]. Nevertheless, better knowledge about recurrence rate and pattern may improve perioperative, intraoperative and postoperative decision-making, e.g., in a tailored individual approach of postoperative surveillance.

Moreover, there are several available prognostic tools and clinical factors which obviously improve the prediction and accurate stratification of patient prognosis [19,36,43]. However, currently published data in this context have focused almost exclusively on clinical characteristics such as age, sex, tumor-related factors, etc., whereas BMI or/and appearance of overweight and obesity is left behind [32,33,34,43]. Therefore, the results of this study were of enormous importance, as we were able to demonstrate that if recording BMI as a prognostic factor in clinical research an independent association between increasing patients BMI and incremental development of recurrence risk following curative liver resection among patients with pCAA was observed.

Additionally, moderate/poor tumor differentiation, positive perineural invasion, positive lymph nodes such as a positive resection margin could be identified as independent prognostic risk factors for worse RFS. Although the main focus of our study was not to discuss mechanistic insights into obesity-related cancer development and recurrence of pCCA, some interesting new aspects were gained.

Thus, we could show that in the absence of differences in initial tumor presentation, perioperative outcome and recurrence pathways between the individual BMI groups, increasing BMI was still associated with an increased risk of recurrence. In numerical terms, it indicates that every unit increase in patient BMI was associated with 8.8% increase in the risk of recurrence. In this context, our study hypothesized that not only classical tumor characteristics exert considerable influence on prognosis in patients with pCCA.

As mentioned previously, obesity lead to of chronic inflammation, the findings of future studies are necessary to elucidate the distinct gene expression patterns as well as genetic alterations in this patient collective. The strength of our study is a relatively large data set on a selected group of patients treated in a high-volume liver tumor center. This contains beside demographics, pathologic and radiologic data as well as short and long-term clinical and oncological information.

The main limitation however is its retrospective character and the small sample size analyzed in the different groups, therefore unexpected bias and missing statistical power cannot completely ruled out. Further, as previous studies have already described, several factors seem to play a role in clarifying the relationship between obesity and cancer survival or prognosis, consisting of methodological limitations in study method and design, reverse causation as well as confounding variables and collider stratification bias [44].

In this context, the assignment of our patients to the respective groups is based solely on the definition of the patients BMI which is commonly used measure of body fatness in studies and clinics. However, the problem is that the BMI does not take into account individual composition of a person’s adipose tissue (visceral and/or subcutan adipose tissue) and lean/muscle tissue.

It is well known that visceral obesity may play an important role in development of different cancers and as such defining obesity by BMI could underestimate the frequency of visceral obesity in this population. This may result in a possibly erroneous assessment of the relationship between obesity and cancer related outcomes.

Finally, our patient population included in the study is not really representative as it only includes patients who underwent curative intent liver resection, and our findings cannot necessarily be extrapolated to those who did not undergo liver resection.

However, this study firstly identified the BMI as a strong prognostic parameter of recurrence and increased recurrence-free survival in patients undergoing liver resection for perihilar cholangiocarcinoma.

## 5. Conclusions

We could show that obesity—expressed as an increased BMI—was an independent prognostic predictor of increased risk of recurrence and subsequent poorer recurrence-free survival following curative intent liver resection of pCCA. In addition, moderate/poor tumor differentiation, positive perineural invasion, positive lymph nodes such as positive resection margins are independent risk factors for recurrence.

Against the background of increasing numbers of patients with pCCA in line with the continuous increase of overweight and obesity all over the world the prognostic role of BMI in the clinical setting in patients with cholangiocarcinoma—in particular in the subset of pCCA—must be reconsidered to refine appropriate diagnostical and therapeutical measurements.

Although the definition of obesity via BMI calculation can be easily performed, the clinical assessment of the risk profile in these patients is inadequate as it does not appropriately reflect body (fat) composition or the ratio of visceral/adipose tissue, body weight and their impact on metabolism, inflammatory and immunity.

Therefore, reliable and practical methods are of enormous importance, especially in cancer survivorship and weight trajectories, which can further advance this field and develop appropriate measures for the integration of BMI in daily clinical practice.

Future research may aim to understand factors that go beyond classical pathological and epidemiological characteristics of pCCA and elucidate mechanism associated with worse outcomes.

## Figures and Tables

**Table 1:** Demographics and baseline characteristics.

Variables	Normal Weight (*n* = 48 Patients)	Overweight (*n* = 33 Patients)	Obese (*n* = 14 Patients)	*p*-Value
Patients	48 (50.5%)	33 (34.7%)	14 (14.7%)	-
Height (cm)	171 ± 8	168 ± 8	168 ± 10	0.097
Weight (kg)	66 ± 8	76 ± 8	99 ± 12	<0.01
Gender				0.89
Male	26 (54.2%)	19 (57.6%)	7 (50%)	
Female	22 (45.8%)	14 (42.4%)	7 (50%)	
Age (years)	62.1 ± 1.7	64.9 ± 1.5	61.3 ± 2.5	0.39
ASA, score (%)				<0.01
1–2	39 (82%)	23 (70%)	5 (36%)	
3–4	9 (18%)	10 (30%)	9 (64%)	
Diabetes mellitus				0.21
Yes	13 (27.1%)	8 (24.2%)	6 (42.9%)	
No	34 (70.8)	22 (66.7%)	6 (42.9%)	
Missing	1 (2.1%)	3 (9.1%)	2 (14.2%)	
Cirrhosis/Fibrosis				0.91
Yes	5 (10.4%)	4 (12.1%)	2 (14.3%)	
No	43 (89.6%)	29 (87.9%)	12 (85.7%)	
Clinical Jaundice				0.79
Yes	38 (79.1%)	27 (81.8%)	10 (71.4%)	
No	10 (20.9%)	6 (18.2%)	4 (28.6%)	
CA 19-9, (U/L)	292.7 ± 101.6	340.2 ± 582.5	338.6 ± 669	0.89
CEA, (ng/mL)	6.2 ± 2.3	2.8 ± 0.8	2.1 ± 0.6	0.31
Peak Bilirubin preoperative (ummol/L)	44.1 ± 6.8	70.8 ± 15.4	33.9 ± 13.1	0.11
Preoperative drainage				0.29
No	7 (14.6%)	6 (18.2%)	4 (28.6%)	
PTCD	7 (14.6%)	2 (6.1%)	0 (0%)	
ERCP	33 (68.8%)	20 (60.6%)	8 (57.1%)	
Both	0 (0%)	2 (6.1%)	1 (7.1%)	
Missing	1 (2.1%)	3 (9.1%)	1 (7.1%)	
Portal vein involvement on imaging				0.693
Yes	12 (25%)	8 (24.2)	2 (14.3)	
No	36 (75%)	25 (75.8)	12 (85.7)	
Bismuth-Corlette classification				0.64
I	2 (4.2%)	2 (6.1%)	2 (14.3%)	
II	4 (8.3%)	2 (6.1%)	2 (14.3%)	
IIIa	6 (12.5%)	4 (12.1%)	1 (7.1%)	
IIIb	7 (14.6%)	3 (9.1%)	2 (14.3%)	
IV	25 (52.1%)	20 (60.6%)	4 (28.6%)	
Missing	4 (8.3%)	2 (6.1%)	3 (21.4)	

**Table 2:** pCCA tumor characteristics.

Variables	Normal Weight (*n* = 48 Patients)	Overweight (*n* = 33 Patients)	Obese (*n* = 14 Patients)	*p*-Value
Tumor size (mm)	41.5 ± 3.8	31.1 ± 3.3	29.5 ± 4.3	0.09
Tumor Differentiation				0.37
Well (G1)	4 (8.3%)	1 (3%)	0 (0%)	
Moderately (G2)	24 (50%)	12 (36.4%)	6 (42.9%)	
Poor (G3)	20 (41.7%)	20 (60.6%)	8 (57.1%)	
AJCC, 8th edition				0.426
I	3 (6.3%)	0 (0%)	2 (5.3%)	
II	21 (43.8%)	22 (66.7%)	9 (64.3%)	
IIIA	2 (4.2%)	1 (3%)	1 (7.1%)	
IIIB	2 (4.2%)	2 (6.1%)	0 (0%)	
IIIC	13 (27.1%)	4 (12.1%)	2 (14.3%)	
IVA	3 (6.3%)	1 (3%)	0 (0%)	
IvB	4 (8.3%)	3 (9.1%)	0 (0%)	
Resection margin status				0.44
Microscopic negative (R0)	36 (75%)	21 (63.6%)	11 (78.6%)	
Microscopic positive (R1)	12 (25%)	12 (36.4%)	3 (21.4)	
Vascular Invasion				0.951
No	35 (72.9%)	23 (69.7%)	10 (71.4%)	
Yes	13 (27.1%)	10 (30.3%)	4 (28.6%)	
Perineural Invasion				0.848
No	8 (16.7%)	7 (21.2%)	3 (21.4%)	
Yes	40 (83.3%)	26 (78.8%)	11 (78.6%)	
Invasion of small lymphatic vessels				0.512
No	12 (25%)	10 (30.3%)	2 (14.3%)	
Yes	36 (75%)	23 (63.6%)	12 (85.7%)	
Metastasic lymph node				0.232
Negative	28 (58.3%)	24 (72.7%)	11 (78.6%)	
Positive	20 (41.7%)	9 (27.3%)	3 (21.4%)	
Metastasic Lymph node				0.555
0	28 (58.3%)	24 (72.7%)	11 (78.6%)	
1–3	15 (31.3%)	7 (21.2%)	2 (14.3%)	
>3	5 (10.4%)	2 (6.1%)	1 (7.1%)	
Harvested Lymph node	4.9 ± 0.5	5.2 ± 0.6	4.1 ± 0.9	0.59
Mean and SEM				
Lymph node				0.67
harvested				
0–5	30 (62.5%)	19 (57.6%)	10 (71.4%)	
>5	18 (37.5%)	14 (42.4%)	4 (28.6%)	

**Table 3:** Operative and postoperative patient characteristics.

Variables	Normal Weight (*n* = 48 Patients)	Overweight (*n* = 33 Patients)	Obese (*n* = 14 Patients)	*p*-Value
Extent of liver surgery				0.27
Wedge resection +	0 (0%)	3 (9.1%)	1 (7.1%)	
Hemihepatectomy right	3 (6.3%)	4 (12.1%)	3 (21.4%)	
Hemihepatectomy left	5 (10.4%)	3 (9.1%)	3 (21.4%)	
Extended hemihepatectomy right	31 (64.6%)	18 (54.5%)	5 (35.7%)	
Extended hemihepatectomy left	9 (18.8%)	5. (15.2%)	2. (14.3%)	
Portal vein resection				0.91
Yes	34 (70.8%)	24 (72.7%)	10 (71.4%)	
No	14 (29.2%)	9 (27.3%)	3 (28.6%)	
Operative time (min)	392 ± 93	417 ± 101	453 ± 116	0.27
Blood loss (ml)	960 ± 320	600 ± 170	490 ± 590	0.22
mean and SEM				
Blood transfusion				0.88
Yes	21 (43.8%)	14 (42.4%)	7 (50%)	
No	27 (56.2%)	19 (57.6%)	7 (50%)	
Hospital stay (days)	23.8 ± 6.3	26.2 ± 5.2	32.8 ± 8.2	0.001
Stay in ICU (days)	5.3 ± 3.3	6.2 ± 6.5	9.5 ± 10.1	0.21
Grade of Complications				0.567
Minor (I/II)	7 (7.3%)	6 (6.3%)	1 (1%)	
Major (>III)	23 (24.2%)	19 (20%)	10 (10.5%)	
Adjuvant chemotherapy				0.02
Yes	4 (8.3%)	9 (27.3%)	5 (35.7%)	
No	44 (91.7%)	24 (72.7%)	9 (64.3%)	

**Table 4:** Risk factors for recurrence after surgery for pCCA on univariate analysis.

Parameters	Median Survival	5 Years RFS	*p*-Value
Age			
≤65	52 (17.9–86.1)	38	0.03
>65	29 (10.1–47.9)	19	
Bismuth Classification			
I/II	60 (5.7–114.2)	43	0.539
III/IV	36 (26.7–45.2)	30	
Gender			0.280
female	37 (22.6–51.3)	30	
male	28 (12.2–43.7)	28	
Residual tumor			
R0	42 (16–76)	41	<0.01
R1	26 (0–57.8)	19	
Tumor differentiation			
Well (G1)	50 (18.7–81.2)	45	<0.01
Moderately/Poor (G2/G3)	18 (1.9–34.4)	12	
Lymph node status			
Negative	40 (24.6–55.4)	46	0.015
Positive	17 (0.1–36.7)	17	
Vascular invasion			
No	40 (10.2–69.7)	40	0.037
Yes	27 (10.1–43.9)	15	
Perineural invasion			
No	45 (11.2–92.3)	68	0.021
Yes	28 (18.5–55.4)	30	
Invasion of small lymphatic vessels			
No	45 (26.3–67.9)	54	<0.01
Yes	28 (18.3–37.6)	25	
Adjuvant chemotherapy			
No	30 (21.7–38.2)	27	0.810
Yes	40 (14.2–65.7)	29	
Preoperative drainage			
No	40 (25.8–54.1)	36	0.478
PTCD	10 (1.6–18.4)	19	
ERCP	36 (13.5–58.4)	25	
AJCC-stage-8th edition			0.03
1	60 (22.4–91.9)	60	
2	50 (27.1–72.9)	38	
3	36 (10.6–61.3)	21	
4	12 (0–38.1)	0	
Tumor diameter			0.919
<30	37 (29.2–44.7)	36	
>30	36 (22.4–49.5)	31	
Perioperative blood transfusion			
Yes	17 (0–40.9)	18	0.023
No	40 (20.3–59.6)	38	

**Table 5:** Multivariate Cox regression analysis of factors associated with recurrence-free **survival**.

Variables	HR	95% CI	*p*-Value
Body mass index	1.08	1.01–1.16	0.021
Moderate and poor tumor grading	2.49	1.2–5.2	0.014
Positive perineural Invasion	2.92	1.02–8.3	0.045
Positive lymph nodes	2.01	1.11–3.65	0.021
Resection margin positive	1.89	1.02–3.4	0.019

## Data Availability

Our database contains highly sensible data which may provide insight in clinical and personnel information about our patients and lead to identification of these patients. Therefore, according to organizational restrictions and regulations these data cannot be made publicly available. However, the datasets used and/or analyzed during the current study are available from the corresponding author on reasonable request.

## Abbreviations

BMI	body mass index
PHC	perihilar cholangiocarcinoma
HPB	hepato-pancreato-biliary
WHO	World Health Organization
RFS	recurrence-free survival
EU	European region
AJCC	American Joint Committee on Cancer
UICC	International Union Against Cancer
CT	computed tomography
MRI	magnetic resonance imaging
MRCP	magnetic resonance cholangiopancreatography
EBD	endoscopic biliary drainage
PTBD	percutaneous transhepatic biliary drainage
PVE	portal vein embolization
CLR	caudate lobe resection
CEA	carcinoembryonic antigen
CA 19-9	cancer antigen 19-9
ASA	American society of anesthesiologists
TNM	tumor node metastasis
ANOVA	analysis of variance
RI	recurrence interval
RFS	recurrence free survival
HR	hazard ratio
CI	confidence interval
HCC	hepatocellular carcinoma
iCCA	intrahepatic cholangiocarcinoma
NASH	non-alcoholic steatohepatitis
NAFLD	non-alcoholic fatty liver disease
eCCA	extrahepatic cholangiocarcinoma
